# Regulation of lipid droplet turnover by ubiquitin ligases

**DOI:** 10.1186/1741-7007-8-94

**Published:** 2010-07-19

**Authors:** Philipp Alberts, Daniela Rotin

**Affiliations:** 1 Cell Biology Program, The Hospital for Sick Children, and Biochemistry Department, University of Toronto, Toronto, Ontario, M5G 1L7 Canada

## Abstract

Mutation of the protein spartin is a cause of one form of spastic paraplegia. Spartin interacts with ubiquitin ligases of the Nedd4 family, and a recent report in *BMC Biology *now shows that it acts as an adaptor to recruit and activate the ubiquitin ligase AIP4 onto lipid droplets, leading to the ubiquitination and degradation of droplet-associated proteins. A deficiency of spartin apparently causes lipid droplets to accumulate.

See research article: http://www.biomedcentral.com/1741-7007/8/72/

## Commentary

The hereditary spastic paraplegias (HSPs) are a heterogeneous group of neurodegenerative disorders that all share progressive lower limb spastic paralysis. Despite their genetic and phenotypic heterogeneity (more than 20 gene loci have been described), all HSPs involve progressive degeneration of axonal tracts of corticospinal motor neurons. These exceptionally long axons, which can reach over a meter in length on their way from the motor cortex in the brain to the spinal cord, represent an extreme example of the challenges faced by a neuron to translate genetic information into cellular function over long distances. Perhaps not surprisingly, genes mutated in different manifestations of HSP affect diverse cellular functions such as cell adhesion, mitochondrial function/energy metabolism and membrane trafficking [1].

The gene for the protein spartin was identified in the search for mutations causing a complicated form of HSP called Troyer syndrome. A single base deletion was identified in the *spartin *gene, causing a frameshift mutation and premature termination of the spartin protein [2]. The amino terminus of spartin encodes a microtubule interacting and trafficking (MIT) domain that is also found in trafficking proteins involved in protein degradation through the endosomal/lysosomal system. Indeed, spartin partly localizes to endosomal structures and appears to facilitate degradation of activated epidermal growth factor receptors, a process that is attenuated in spartin-depleted cells [3,4].

Recent work [4,5], including a study by Hooper *et al. *[6] published in *BMC Biology*, has now discovered an unexpected new function for spartin. All three groups describe recruitment of spartin to newly formed lipid droplets induced by oleic acid treatment [4-6]. Lipid droplets are dynamic lipid-storage organelles that are formed when there is a constant exogenous supply of fatty acids. Upon a change in metabolic conditions, stored neutral fats can be mobilized for lipolysis and can thereby contribute to the energy homeostasis of the cell [7]. Interestingly, spartin's localization to lipid droplets depends on the carboxy-terminal part of the protein that is deleted in Troyer syndrome [4,6], which suggests that defects in lipid-droplet formation and dynamics in neurons could underlie this syndrome.

What might be the role of spartin, a protein with no known catalytic activities, in lipid-droplet dynamics? Spartin is mono-ubiquitinated and possesses a proline (P)-rich PY-motif (PPxY; Y is tyrosine and x is any amino acid), often found in proteins that interact with ubiquitin ligases of the Nedd4 family. Indeed, spartin was shown to interact with the Nedd4-family E3 ligases AIP2, AIP4 (Itch) and AIP5 [5]. Hallmarks of these ubiquitin ligases are an amino-terminal C2 domain involved in lipid-protein interaction, two to four WW domains responsible for substrate recognition via the PY motif, and a carboxy-terminal catalytic HECT domain. Through their ubiquitin ligase activity, members of the Nedd4 family are involved in many biological processes, via proteasome-dependent degradation of cytosolic targets, regulation of endocytosis and lysosomal degradation of surface receptors and channels, or by aiding egress of viral particles through the endosomal sorting complexes required for transport (ESCRT).

**Figure 1:**
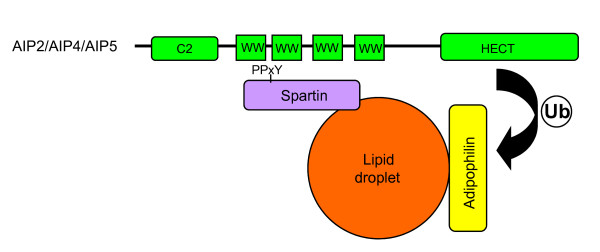
**Spartin associates with lipid droplets and possesses a PY motif that recruits the ubiquitin ligase AIP4 (or other Nedd4-family ubiquitin ligases) via the AIP4 WW domains**. Via its catalytic HECT domain, AIP4 promotes the ubiquitination of lipid-droplet-associated proteins, such as adipophilin, leading to regulation of turnover of the lipid droplets. Thus, spartin functions as an adaptor protein to promote ubiquitination (Ub) of lipid-droplet-associated proteins. In the absence of spartin, the catalytic activity of AIP4 is inhibited by intramolecular interactions (not shown).

Hooper *at al. *[6] set out to analyze the significance of the interaction of spartin and the ubiquitin ligase AIP4. Surprisingly, despite binding to AIP4 via its PY motif, spartin was not a substrate for AIP4-mediated ubiquitination. These observations are in agreement with those of Edwards *et al. *[4], who were unable to detect changes in spartin ubiquitination levels upon overexpression or knockdown of AIP4 or AIP5. Yet, both these ubiquitin ligases interact with spartin. Instead, spartin seems to play a role in the localization of AIP4. Whereas overexpressed AIP4 shows a diffuse cytosolic distribution, coexpression of wild-type spartin promotes recruitment of AIP4 to lipid droplets; mutations of the PY motif in spartin that disrupt binding to AIP4 also disrupt spartin's ability to recruit AIP4, without affecting spartin's own localization to the droplets [6]. These findings suggest that spartin binds to AIP4 through its PY motif to mediate spatial regulation of ubiquitination (Figure 1).

Hooper *et al. *[6] therefore investigated the involvement of spartin in the ubiquitination of known ubiquitin ligase substrates on the lipid-droplet membrane. One of these is adipophilin, and it and the proteins peripilin and TIP47 form the PAT family of peripherally associated membrane proteins highly enriched on lipid droplets. Adipophilin plays a role in lipid droplet turnover, where it associates with lipid droplets at early stages of droplet biogenesis, but undergoes ubiquitin-induced degradation during mobilization of stored lipids. Thus, ubiquitination of lipid--droplet-associated proteins has great regulatory potential [7]. Indeed, on knockdown of spartin, Hooper *et al. *[6] found that the ubiquitination of adipophilin is greatly reduced, supporting a role for spartin in the recruitment of ubiquitin ligases to these lipid bodies (Figure 1). Together with the earlier reports, their findings support a role for ubiquitination in the dynamics of lipid droplets, as depletion of endogenous spartin leads to apparent accumulation of lipid bodies in cells [4,6].

Spartin is therefore an addition to the list of mammalian adaptor proteins interacting with and regulating Nedd4-family ligases. Other membrane-expressed adaptors for AIP4 and AIP2 containing PY motifs have been described recently. For example, Ndfip1 binds to AIP2 through two PY motifs and mediates ubiquitination of the membrane-bound iron transporter DMT1 [8]. Similarly, AIP4 binds to its target JunB in T lymphocytes through the same adaptor protein, Ndfip1, to allow ubiquitination of JunB and containment of JunB-dependent signaling. AIP4-knockout mice and Ndfip1-knockout mice both develop severe autoimmunity [9], making a strong case for the importance of adaptors in enabling substrate-ligase interaction, at least in some scenarios.

In addition, spartin binding to AIP4 seems to have a direct effect on the catalytic activity of the ubiquitin ligase. Intramolecular inhibition has been described for AIP4. The carboxy-terminal HECT domain folds back onto the WW domain by binding to a proline-rich region amino-terminal to the WW domains, greatly inhibiting AIP4's catalytic activity. Phosphorylation events in the proline-rich region disrupt the intramolecular interactions and greatly stimulate the catalytic activity of AIP4 [10]. A similar autoinhibitory mechanism has been suggested to restrict the catalytic activity of Nedd4-2, which could be relieved by Nedd4-2 binding to a PY motif-containing *bona fide *substrate (ENaC [11]), and Smurf2, in which the C2 domains bind to and inhibit the HECT domain [12]. Via its PY motif, spartin binds to the WW domains of AIP4 with high affinity, which Hooper *et al. *[6]now show can disrupt the intramolecular interaction between the HECT and WW domains, and increase AIP4's catalytic activity.

Thus, a scenario is emerging in which spartin recruits and activates Nedd4-family ligases for ubiquitination and degradation of lipid droplet proteins such as adipophilin. In response to extracellular changes, cells are well known to utilize the ubiquitin/proteasome system to control levels of transcription factors, cell-cycle proteins or membrane receptors. The novel findings on spartin suggest a role for ubiquitination in the turnover of an organelle - the lipid droplet.

Given the original description of mutated spartin as the cause of a particularly devastating form of HSP, an important question to be investigated is the relevance of spartin's association with lipid bodies to the development of HSP. As Hooper *et al. *discuss, another gene product, SPG17 or seipin, involved in a different variant of HSP, is implicated in lipid-droplet maintenance in yeast. Analysis of the specific role of spartin in neurons should yield a better understanding of the unexpected connection between lipid-droplet dynamics and neuronal degeneration as seen in Troyer syndrome.
